# The effect of smartphone dependence on learning burnout among undergraduates: the mediating effect of academic adaptability and the moderating effect of self-efficacy

**DOI:** 10.3389/fpsyt.2023.1155544

**Published:** 2023-09-06

**Authors:** Chunmei Chen, Yuanyi Shen, Fanghao Xiao, Jianchao Ni, Yujie Zhu

**Affiliations:** ^1^Teachers College, Jimei University, Xiamen, Fujian, China; ^2^School of Aerospace Engineering, Xiamen University, Xiamen, Fujian, China; ^3^College of Computer and Information Engineering, Xiamen Institute of Technology, Xiamen, Fujian, China; ^4^School of Aerospace Engineering, Xiamen University, Xiamen, Fujian, China; ^5^School of Marine Culture and Law, Jimei University, Xiamen, Fujian, China

**Keywords:** mobile phone dependence, learning burnout, academic adaptability, self-efficacy, mediating effect, moderating effect

## Abstract

**Introduction:**

Smartphone dependence is closely related to the physical and mental health development of undergraduates and their learning. The purpose of this study was to explore the relationship between smartphone dependence, academic adaptability, self-efficacy and learning burnout among undergraduates and its underlying mechanisms.

**Methods:**

The study was conducted on 2,110 undergraduates using the Smartphone Dependence Scale, the Undergraduates Learning Adjustment Scale, the Learning Burnout Undergraduates Scale and the Self-Efficacy Scale to develop a mediation model and a moderation model.

**Results:**

The findings of this study revealed that (1) smartphone dependence significantly negatively predicted academic adaptability; (2) academic adaptability significantly negatively predicted learning burnout; (3) smartphone dependence significantly positively predicted learning burnout; (4) academic adaptability partially mediated the effect of smartphone dependence on learning burnout; (5) self-efficacy played a moderating role in the effect of academic adaptability on learning burnout.

**Conclusion:**

These findings can help researchers and educators better understand the underlying mechanisms between smartphone dependence and learning burnout in undergraduates.

## 1. Introduction

Mobile phones are portable handheld devices that can support people to stay in touch with family, friends, etc. through voice calls regardless of time and place (1). Smartphones not only have the communication function of cell phone, but also multimedia playback, game entertainment, Internet browsing, e-mail, social network, map navigation and other functions of a computer. This is because smartphones are a combination of cell phone and handheld computer (2). Smartphones have features such as higher performance ratios, greater automation for the users, and the ability to improve and control information accordingly (3). The first smartphone was developed in 1993. In 2011, smartphone ownership among adults in the United States reached 35% (4). In 2018, smartphone ownership *per capita* in South Korea reached 90% (5). In China, the early twenty-first century a new wave of rapid expansion in the telecommunication industry increased the availability of smartphones. The cost of owning and using a smartphone fell due to the “two-pronged effect” of diversifying smartphone products, visualizing and making data costs more affordable (6). According to the China Internet Network Information Center (7), as of June 2022, the number of mobile Internet users in China reached 1.047 billion and for instant messaging users was 1.027 billion. Smartphones have a huge appeal to the young population. Not only can they make calls and send text messages, but they can also access the Internet, make videos, and record information, among other things (8). In the era of the new coronavirus outbreak, the flexible and diverse applications of smartphones have made their communication function increasingly important (9). As the main carrier of the mobile Internet, smartphones have various functions such as entertainment, socialization, and shopping, which have facilitated human life but also brought about corresponding usage issues, one of which is smartphone dependence symptoms (10). Park (11) argues that excessive reliance on smartphones fits the classic “behavioral addiction.” Psychiatrists regard it as an “overuse” behavior that has various negative effects on the individual’s physical and mental health and learning progress. Psychologists see it as an “addictive behavior.” As a remarkable hobby and habit, it causes excitement and pleasure to the users. Smartphone dependence can also lead to communication barriers, cognitive barriers to learning, interpersonal barriers, and fragmentation of social relationships, which have become important factors affecting students’ academic development (12, 13). Furthermore, Horwood and Anglim (14) note that the phenomenon of smartphone dependence has become a major problem that seriously affects the psycho-social development of university students or adolescents. It may have a negative impact on university students’ compliance in group norms, psychological needs satisfaction, and emotional transference.

### 1.1. The relationship between smartphone dependence and academic adaptability

The level of academic adaptability has a significant impact on the academic development of college students (15). Unstable levels of academic adaptability may lead to a decrease in students’ interest in learning, emotional instability, and even a decrease in their academic performance, thus affecting their healthy physical and mental development (16). Students’ academic adaptability refers to the ability of students to adapt to the learning environment and complete their learning tasks after entering university. Students’ ability to plan and track their studies is an important manifestation of their academic adaptability (17). This ability to plan and track their studies has been seriously undermined by smartphones (18). Smartphone dependence symptoms have become a common phenomenon among the university student population. The huge amount of information and overloaded entertainment functions of smartphones tend to negatively affect students’ academic adaptability (19, 20). Lee and Cho (21) identify that smartphones encourage the use of email and checking of instant messages during class and they distract students while studying or doing homework. Smartphone dependence and academic adaptability are significantly and negatively related. Huang and Guo (22) come to similar conclusions. They note that smartphone dependence inversely predicts the level of academic adaptability and is ultimately reflected in academic performance.

*H1*: There is a significant effect of smartphone dependence on academic adaptability.

### 1.2. The relationship between academic adaptability and learning burnout

Emotional exhaustion, depersonalization, and academic ineffectiveness are the three dimensions of learning burnout, which are mainly manifested by students’ lack of active participation in class, lack of interest in the course, and avoidance of class discussions (23). According to Li and Wang (24), learning burnout is caused by chronic academic stress or academic load and energy drain. Students become progressively less motivated by school assignments and activities, which leads to apathy and detachment resulting in the unexpected phenomenon of students’ negative attitude toward their studies. In addition, Li and Wang explore nine aspects of university students’ academic adaptability in terms of fear of failure, test anxiety, test preparation, and study efficiency and point out that students with poor academic adaptability tend to experience more problems with learning burnout (24). Xie et al. (25) conducted a study of medical students and found that academic adaptability had a significant negative predictive effect on learning burnout. That is, students with high academic adaptability have low levels of learning burnout. It has been established that academic adaptability is negatively correlated with learning burnout (26). According to Xie and Derakhshan (27), academic adaptability is a long-term self-regulatory process, and learning burnout is also a constantly changing psychological variable. Academic adaptability will inevitably have an impact on learning burnout. If students do not adapt quickly to their studies and life, they become out of place, which can easily lead to academic disabilities and learning burnout. In summary, hypothesis H2 is proposed.

*H2*: There is a significant effect of academic adaptability on learning burnout.

### 1.3. The relationship between smartphone dependence and learning burnout

Overall smartphone use can have an impact on students’ academic achievement. Students who are not dependent on their phones are more likely to make progress in their participation in equivalent courses. There is a positive relationship between smartphone dependence and learning burnout (28). Zhang et al. (29) find that the higher the level of smartphone dependence, the less will power students have to resist and control the temptation of smartphones, the slower they respond to the teacher, and the more likely they are to fall into learning burnout, such as lack of interest in academic life, lethargy, avoidance, and even indifference. Related studies have shown that smartphone dependence is positively correlated with learning burnout. That is, the higher the level of smartphone dependence among university students, the more likely they are to experience learning burnout (30, 31). Zhou (32) put forward a similar conclusion. She also emphasizes that smartphone dependence can have an impact on learning burnout both directly and indirectly through psychological capital. In summary, hypothesis H3 is proposed.

*H3*: There is a significant effect of smartphone dependence on learning burnout.

### 1.4. The mediating effect of academic adaptability

Tanil and Yong (33) find that excessive use of smartphones will have a negative impact on university students’ learning and memory, increase their learning cognitive load, and then affect students’ ability to accept and adapt to learning tasks. Choe and Yu (34) point out that the more students rely on their smartphones, the more difficulty they will have in adapting to their study life, especially in maintaining their interest and academic performance, and complying with classroom rules. Studies have confirmed that smartphone dependence is negatively associated with academic adaptability (35). In addition, Xie et al. (25) argues that the lower a student’s academic adaptability, the less self-control they have and the lower the sense of personal accomplishment they receive. They develop a greater sense of ineffectiveness and powerlessness in their learning, which prevents them from staying engaged in their studies. These students are more likely to develop learning burnout ideas such as avoiding learning, giving up learning, and slacking off on learning under the effect of depression, boredom and anxiety. Many studies have found that there is a significant negative correlation between academic adaptability and learning burnout (25, 26, 36). In summary, hypothesis H4 is proposed.

*H4*: Academic adaptability mediates the relationship between smartphone dependence and learning burnout.

### 1.5. The moderating effects of self-efficacy

Self-efficacy is a theory developed by Bandura (37), a leading American psychologist, in 1977. Self-efficacy is the expectation that individuals believe they are capable of performing a certain behavior or activity (38). Bandura’s self-efficacy theory suggests that self-efficacy can influence an individual’s objective behavior in a social context. Self-efficacy arises from self-reflection and is goal-oriented and process-evaluative, which remains highly consistent with current research on human object behavior (39). Self-efficacy is one of the prerequisites for meaningful learning (40). It cannot be enhanced without task-based effort and persistence (41). Success promotes the acquisition of self-efficacy and, correspondingly, failure diminishes self-efficacy (42). Chronic low self-efficacy can easily lead students into a vicious cycle of self-doubt, self-assumption and self-denial. In a prolonged stressful environment, students are prone to the dilemma of depleted learning resources (43). The lower a student’s self-efficacy, the less resilient they are to learning (44). Increased self-efficacy can help students accumulate positive, favorable outcome expectations. Students with high self-efficacy are able to shed the baggage of intimidation when dealing with difficult learning tasks and always integrate into a complex environment with a relatively optimistic and fulfilling state, thus improving learning adaptability (45). Taufiq-Hail et al. (46) state that self-efficacy positively predicts the level of academic adaptability. Self-efficacy is correlated with the presence of learning adaptation. Furthermore, Mostert and Pienaar (47) describe that lack of self-efficacy is one of the important factors causing learning burnout. Wipawayangkool et al. (48) argue that self-efficacy provides a buffer mechanism for students to deal with stressors, and the inability to handle stress rationally is an important reason for the phenomenon of burnout. Self-efficacy negatively predicts the phenomenon of burnout (49). Students’ low self-efficacy due to failure to meet expectations or lack of coping strategies can cause them to become apathetic to learning and eventually deplete their energy. In extreme cases, low self-efficacy may lead directly to students leaving school (50).

**Figure 1:**
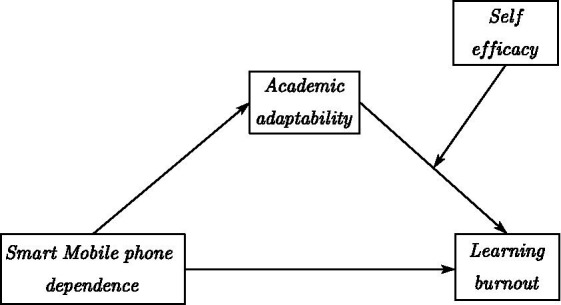
Theoretical model.

**Figure 2:**
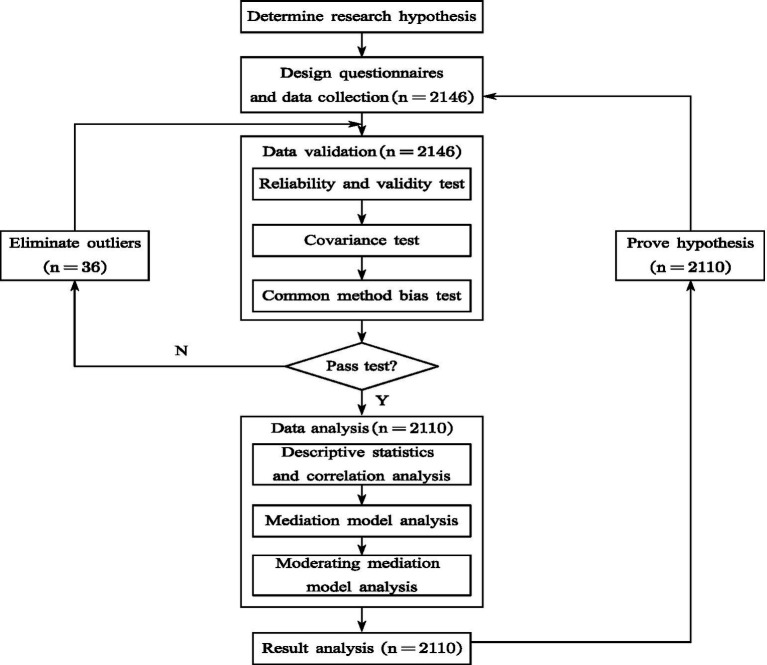
The study flow. When the method of eliminating outliers is invalid, research hypothesis and questionnaire redesign is considered.

This study constructed a mediation model and a regulation model to investigate the mechanism of smartphone dependence on learning burnout in a group of university students, thus to provide new ideas for university students to improve their learning motivation. The theoretical model and the study flow chart are shown in Figures 1, 2, respectively.

## 2. Methods

### 2.1. Data sources and sample characteristics

A total of 2,145 full-time students from different levels of universities and regions of China, such as Xiamen University, Jimei University, Xiamen Institute of Technology, Lanzhou University, Shanghai University etc. were selected as the subjects of the study using the convenience sampling method. Informed consent was obtained by providing the participants with a clear explanation of the purpose and nature of the survey, the procedures involved, the potential risks and benefits, and their rights as study participants. Before completing the questionnaire, participants were given the opportunity to ask questions. Before completing the survey, participants were informed that the questionnaire would be anonymous and free from any commercial factors. Completion of the questionnaire was on a voluntary basis. Participants were interested in filling out this questionnaire because it dealt with issues such as smartphone dependence, academic adaptability, learning burnout, and self-efficacy, all of which were highly relevant to their daily lives. Additionally, participants were motivated to participate in this study in order to provide valuable information for researchers who sought to gain a deeper understanding of these issues and developed effective solutions. After determining the validity of the recovered questionnaire by checking the answers to trap questions and reverse items, the inconsistent questionnaires were eliminated. A total of 2,110 valid questionnaires were obtained, and the effective recovery rate was 98.33%. The sample was well distributed in terms of demographic variables, which was representative and could better meet the needs of this study. There were 1,080 male students (51.18%) and 1,030 female students (48.82%); 706 science students (33.46%), 722 engineering students (34.22%), 580 arts students (27.49%), and 102 other students (4.83%). Informed consent was obtained from the subjects before the survey was conducted.

### 2.2. Research tools

#### 2.2.1. Smartphone dependence questionnaire

Drawing on the Smartphone Dependence Questionnaire developed by Toda et al. (51), the questionnaire was adapted to the actual situation of university students and consists of 20 questions. The items are as follows “I would rather lose my wallet or purse than my mobile phone.” “Without thinking, I check my phone for email or voice mail even when it has not rung.” “When I am riding on a train or in similar situations, I tend to handle my mobile phone.” etc. The full 20 questions can be found in Supplementary Appendix 1. All were rated on a 5-point Likert scale, with higher scores representing higher agreement with the question. The KMO value of the scale was 0.931, which means the study data were well suited for extracting information. The Cronbach α coefficient of the scale was 0.902 that the scale had good consistency and valid measurement results. The 20 items were summed and averaged to obtain the variable of learning burnout after reversing the scores of the reverse questions, with higher scores indicating a higher degree of smartphone dependence.

#### 2.2.2. Learning adjustment scale

The Undergraduates Learning Adjustment Scale developed by Feng et al. (52) was used, and the questionnaire was adjusted according to the actual situation of university students. Forty four questions were included in the scale. Questions such as “I have my own study methods and plans, and I can put them into practice.” “My independence has increased significantly since I went to university.” “I do not adapt to the work schedule of the university.” etc. A copy of questionnaire can be found in Supplementary Appendix 2. All were rated on a 5-point Likert scale, with higher scores indicating higher agreement with the question. The KMO value of the scale was 0.943. This means that the study data were well suited for extracting information. Moreover, the Cronbach α coefficient of the scale was 0.869 that the scale had good consistency and validity of the measurement results. The 44 question items were summed and averaged to obtain the variable of academic adaptability after reversing the scores of the reverse questions, with higher scores indicating a higher degree of academic adaptability.

#### 2.2.3. Learning burnout scale

The Learning Burnout Undergraduates Scales (LBUS) developed by Wang (53) was used and adapted to the actual situation of university students. The scale consists of 20 questions, such as “I feel tired when I wake up early in the morning and think about the day of study,” “I feel exhausted after a whole day of study,” “I want to study but I feel that it is boring” etc. A copy of questionnaire can be found in Supplementary Appendix 3. All were rated on a 5-point Likert scale, with higher scores representing higher agreement with the question. The KMO value of the scale was 0.906 and it means that the study data were well suited for extracting information. Moreover, the Cronbach α coefficient of the scale was 0.824 that the scale had good consistency and valid measurement results. The 20 items were summed and averaged to obtain the variable of learning burnout after reversing the scores of the reverse questions, with higher scores indicating higher levels of learning burnout.

#### 2.2.4. Self-efficacy scale

The self-efficacy scale developed by Schwarzer et al. (54) was used and adapted to the actual situation of university students. The scale consists of 10 questions with items such as “I can always solve problems if I try my best,” “I am confident that I can cope effectively with anything that comes up,” “If I put in the necessary effort, I can definitely I can solve most problems if I put in the necessary effort,” etc. A copy of questionnaire can be found in Supplementary Appendix 4. All were rated on a 5-point Likert scale, with higher scores indicating higher agreement with the question. The KMO value of the scale was 0.935 and it means that the study data were very suitable for extracting information. Moreover, the Cronbach α coefficient of the scale was 0.914 that the scale had good consistency and the measurement results were valid. The 10 items were summed and averaged to obtain the variable of self-efficacy after reversing scores of the reverse questions, with higher scores indicating a higher sense of self-efficacy.

### 2.3. Data processing

SPSS 26.0 (55) was used for descriptive statistics and Pearson correlation analysis. To ensure the accuracy of the results, the variance inflation factor (VIF) method was used for covariance testing (if VIF > 10, it means that there is a serious covariance problem between the variables and the corresponding variables need to be excluded). Model 4 and Model 14 in the process plug-in prepared by Hayes (55) was used for moderated mediation effect analysis (56, 57) and the significance of the mediation effect was tested using the bias-corrected percentile Bootstrap method. If the 99% confidence interval does not contain a value of 0, it is considered statistically significant. In addition, to avoid bias in the moderating effects, all variables were standardized beforehand.

## 3. Research results

### 3.1. Common method bias test

Common method bias occurs when both the independent and dependent variable are captured by the same response method, and the consequences of common method bias can be detrimental to the validity of the study (58). For example, the instructions provided by the questionnaire administered by the researchers for data collection may influence the answers provided by different respondents in the same general direction, resulting in indicators with some common variation (59). Therefore, it is necessary to analyze common method bias. Harman’s Single-Factor Test is considered a practical method widely used in psychological empirical research (60). In such an analysis process, the variance variation caused by the common method bias is not significant when the cumulative percentage of the first component is below 40%. The issue of common method bias may arise when data are collected using the self-report method. The Harman single-factor test was used to test for common method bias. The results showed that there were four factors with a characteristic root greater than one, and the total variance explained by the first common factor was 35.63%, which was less than the critical value of 40%. Thus, there was no common method bias in the data of this study.

### 3.2. Descriptive statistics and correlation analysis of each variable

Four variables, including learning burnout, academic adaptability, smartphone dependence and self-efficacy, were analyzed for correlation. Pearson correlation coefficient test was used considering that the main variables were continuous variables. The results showed that there was a significant correlation between the four variables of smartphone dependence, academic adaptability, learning burnout and self-efficacy. The results were shown in Table 1.

**Table 1:** Correlation analysis among the variables.

	Mean standard	Deviation	Learning burnout	Academic adaptability	Smartphone dependence	Self-efficacy
Learning burnout	2.856	0.501	1			
Academic adaptability	3.411	0.419	−0.666**	1		
Smartphone dependence	2.848	0.716	0.274**	−0.234**	1	
Self-efficacy	3.292	0.645	−0.318**	0.372**	0.057**	1

**p < 0.01.

### 3.3. Testing the mediating effect

Model 4 (Model 4 is a simple mediation model) in the SPSS macro developed by Hayes (55) was used to test the mediating effect of academic adaptability in the relationship between smartphone dependence and learning burnout. The results were shown in Tables 2, 3. The positive predictive effect of smartphone dependence on learning burnout was significant (*B* = 0.192, *t* = 13.075, *p* < 0. 001), and hypothesis H3 was verified. Moreover, when the mediating variable was put in, the positive predictive effect of smartphone dependence on learning burnout was still significant, but the effect value was significantly lower (*B* = 0.087, *t* = 7.562, *p* < 0. 001). The negative predictive effect of academic adaptability on learning burnout was significant (*B* = −0.760, *t* = −38.640, *p* < 0.001), and hypothesis H2 was verified. The negative predictive effect of smartphone dependence on academic adaptability was significant (*B* = −0.137, *t* = −11.060, *p* < 0.001), and hypothesis H1 was verified. In addition, the upper and lower limits of the bootstrap 95% confidence interval for the direct effect of smartphone dependence on learning burnout and the mediating effect of academic adaptability did not contain 0 (see Table 3), indicating that the mediating effect existed and was partially mediated, and hypothesis H4 was verified.

**Table 2:** Mediated model test for academic adaptability.

Regression equation (*N* = 1,061)		Fitting indicator			Coefficient significance	
Outcome variables	Predictor variables		Adjustment	Value	
Learning burnout	Constant	0.075	0.075	170.947***	2.311***	53.718
	Smartphone dependence				0.192***	13.075
Academic adaptability	Constant	0.055	0.054	122.313***	3.802***	104.363
	Smartphone dependence				−0.137***	−11.060
Learning burnout	Constant	0.459	0.458	892.487***	5.201***	63.637
	Smartphone dependence				0.087***	7.562
	Academic adaptability				−0.760***	−38.640

***p < 0.001.

**Table 3:** Decomposition of total effect, mediated effect and direct effect.

	Effect value	95% BootCI upper and lower limits	Effect ratio
Total effect	0.192	0.163 ~ 0.220	
Mediated effect	0.104	0.122 ~ 0.182	54.5%
Direct effect	0.087	0.065 ~ 0.110	45.5%

**Figure 3:**
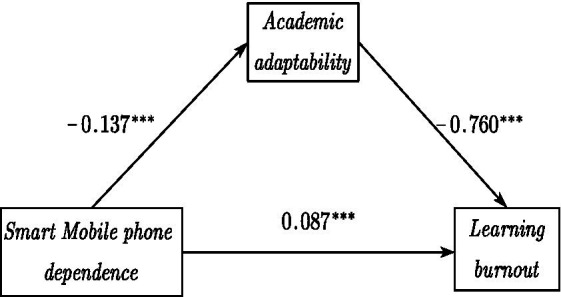
Intermediary model: effect values. ***p < 0.001.

The data results indicated that smartphone dependence significantly and positively predicted learning burnout; academic adaptability significantly and negatively predicted learning burnout, and that smartphone dependence is able to predict learning burnout through the mediating effect of academic adaptability. The mediation model was shown in Figure 3.

### 3.4. Moderating effect test

Again, Model 14 in the SPSS plug-in macro PROCESS prepared by Hayes (55) was used with academic adaptability as the independent variable, learning burnout as the dependent variable, and self-efficacy as the moderating variable. The results were shown in Table 4, and the moderating effects were divided into three models, with Model 1 including the independent variable (adaptability). Model 2 added the moderating variable (self-efficacy) to model 1, and model 3 added the interaction term (the product term of the independent and moderating variables) to model 2. The results showed that academic adaptability significantly and negatively predicted learning burnout (*β* = −0.759, *t* = −36.518, *p* < 0.001), and the interaction term between adaptation and self-efficacy showed significance (*β* = −0.209, *t* = −8.052, *p* = 0.000 < 0.05). These implied that when the moderating variable (self-efficacy) was at different levels, the magnitude of the effect of academic adaptability on the impact of learning burnout was significantly different. What’s more, this moderation was negative.

**Table 4:** Moderating effect test.

Regression equation (*N* = 1,061)		Fitting indicator			Coefficient significance	
Outcome variables	Predictor variables		Adjustment	Value	
Learning burnout	Constant	0.444	0.444	1682.924***	2.856***	351.377
	Academic adaptability				−0.795***	−41.023
Learning burnout	Constant	0.450	0.449	860.850***	2.856***	353.123
	Academic adaptability				−0.759***	−36.518
	Self-efficacy				−0.063***	−4.691
Learning burnout	Constant	0.466	0.465	612.901***	2.877***	343.121
	Academic adaptability				−0.715***	−33.792
	Self-efficacy				−0.059***	−4.438
	Academic adaptability* Self-efficacy				−0.209***	−8.052

***p < 0.001.

**Figure 4:**
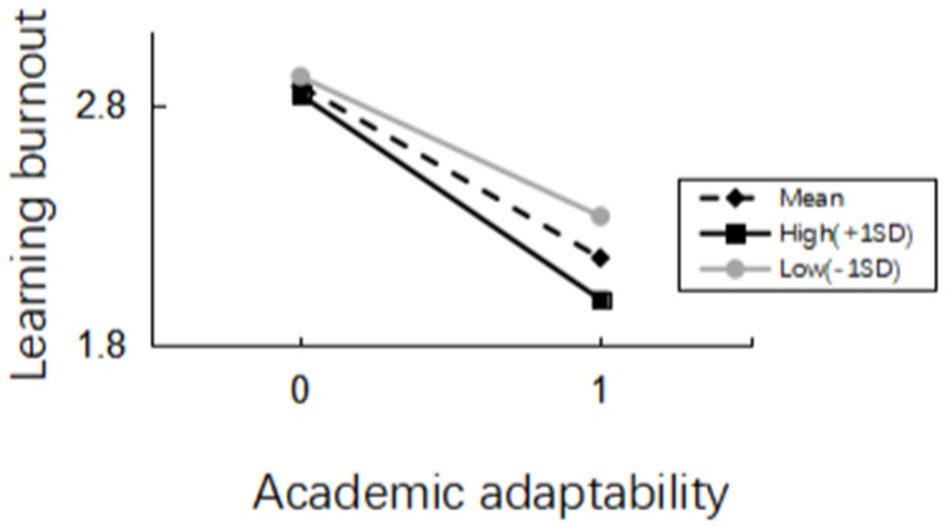
Moderating effects of mean, high and low levels of self-efficacy.

To test whether this pattern of moderating effects was consistent with the hypothesis, we followed Aiken and West’s (61) suggestion of a high subgroup with self-efficacy scores above the mean plus one standard deviation, below the mean minus one standard deviation for the low subgroup. The moderating effects of different self-efficacy were shown in Table 5 and their simple slope plots were shown in Figure 4. The negative predictive relationship between academic adaptability and learning burnout was stronger in the high self-efficacy group (simple slope = −0.850 *p* < 0.001) and weaker in the low self-efficacy group (simple slope = −0.581, *p* < 0.001), and hypothesis H5 was supported.

**Table 5:** Moderating effect of self-efficacy.

Level of moderating variable	Regression coefficient	Standard error	*t*	*p*	95% CI	
Mean	−0.715	0.021	−33.792	0.000	−0.757	−0.674
High level (+1SD)	−0.850	0.023	−36.329	0.000	−0.896	−0.804
Low level (−1SD)	−0.581	0.030	−19.266	0.000	−0.640	−0.522

## 4. Discussion

### 4.1. The effect of smartphone dependence on academic adaptability

The results of this study showed that smartphone dependence negatively predicted academic adaptability, i.e., the higher the level of smartphone dependence, the lower the academic adaptability of the university student group. Conversely, university students with low smartphone dependence had higher academic adaptability. In the study, a significant effect of smartphone dependence on academic adaptability, was verified. This was consistent with the findings of related studies. Studies have found that smartphone dependence negatively affects attention (21) and also leads to impairment of cognitive functioning and emotional/mood states in university students. They are unable to avoid using their phones in class, which distracts them and interferes with their subsequent cognitive tasks (33). As a result, university students’ academic adaptability is affected. Students with a higher level of smartphone dependence have lower academic adaptability, lack planning for their future development, and are unable to use their time wisely. They are overly addicted to the virtual world of smartphones and unable to extricate themselves. In a time crunch, it is difficult for individuals to carry out effective learning and adaptation behavior (62). Gutiérrez-Puertas et al. (63) also find that smartphone dependence negatively affects nursing students’ course work, leading to poor concentration in class and inability to complete off-course study tasks in a timely manner, thus affecting academic performance. Smartphone dependence among university students means that they spend most of their time using various applications on their phones and they have less time and energy to complete their studies, which creates a challenge for them to adapt to university studies.

### 4.2. The effect of academic adaptability on learning burnout

The results of this study showed that academic adaptability negatively predicted learning burnout, i.e., university students with stronger academic adaptability had lower levels of learning burnout. Conversely, university students with low academic adaptability had higher levels of learning burnout. Our study found a significant effect of academic adaptability on learning burnout. This is consistent with the conclusion reached by Chen and Lu (64). They find that students who are more adaptive in their learning experience more psychological adjustment in the process of completing their learning tasks. Students are able to positively adapt to the demands of the learning environment, stimulate their maximum learning potential, and are more likely to achieve high levels of achievement and satisfaction through their hard work. This makes them less likely to feel irritability, anxiety, fatigue and other aspects of learning burnout, and they can adjust in time even if their learning status fluctuates (64). Moreover, they are able to actively develop solutions for adverse situations. These students tend to better adapt to the academic pressures of school and enjoy their studies, and are less likely to suffer from learning burnout (65). Students with high level of adaptability are able to reflect on and summarize their personal behavior after encountering setbacks. They can cope with learning burnout and stress by taking control of their lives (66). As a result, they are better able to complete their academic tasks and achieve better academic results, thus gaining self-confidence and motivating them to study further.

### 4.3. The effect of smartphone dependence on learning burnout

The results of this study showed that smartphone dependence positively predicted learning burnout, i.e., the higher the level of smartphone dependence of the university student group, the higher the level of learning burnout, and vice versa. A number of scholars have reached similar conclusions. Our study also found a significant effect of smartphone dependence on learning burnout. According to Li et al. (30), students with high levels of smartphone dependency tend to spend the vast majority of their time on their phones for self-entertainment and recreation. Many students further reduce their real-life emotional needs due to the pleasure and satisfaction of interpersonal relationships they acquire from the virtual world of their smartphones. Thus, such students gradually lose the desire to express themselves and are not interested in anything beyond their smartphones, including learning tasks, course requirements, and other academic matters. Li et al. (67) hold a similar view. They state that excessive use of smartphones by university students can lead to insufficient study time to maintain academic progress. At the same time, the high level of addiction to smartphone use also increases emotional exhaustion and depletes individuals’ energy, which leads to learning burnout. Zhang et al. (31) point out that it is difficult for students who are addicted to smartphones to escape from the online world in their phones. The incoming and outgoing text messages and software alerts can attract students’ attention, leading to a decrease in their enthusiasm and motivation for learning tasks, which in turn affects their academic performance. Poor academic performance, in turn, can cause students to lose interest in learning, which can lead to learning burnout. The higher the degree of smartphone dependence of university students, the less interest and motivation they have in studying, and thus the more likely they are to develop learning burnout.

### 4.4. Mediating effect of academic adaptability

The results of this study showed that academic adaptability partially mediated the relationship between smartphone dependence and learning burnout. That was, the higher the level of smartphone dependence, the lower the academic adaptability and the higher the level of learning burnout in the university population. Academic adaptability mediates the relationship between smartphone dependence and learning burnout. It is worth emphasizing that the use of smartphones does not always interfere with students’ academic adaptability. Some university students also use smartphones in the classroom to help them understand the lessons. The main point here is the negative impact of students’ excessive and irrational use of smartphones on their learning adaptation. According to Hartley et al. (68), students who frequently multi-tasked on their smartphones have lower levels of academic adaptability. Students with more severe symptoms of smartphone dependence are unable to manage study time and focus, and tend to give up learning outcomes at hand early when faced with relatively difficult study tasks. In contrast, students with a lower level of smartphone dependence are able to have a higher level of self-regulated study management. They are able to curb the desire of excessive smartphone use to the greatest extent, and adjust their study status in a timely manner, thus improving their academic adaptability in terms of study arrangement, study plans, and study habits (22). Students who are more adaptable to learning are able to adapt their learning strategies to changing realities and strive to balance their life between study and personal life. Both this and previous studies have found that this group of students could maximize their sense of accomplishment in academic life by combining work and play, and do not easily give up or escape, thus reducing the occurrence of burnout (69). These students are able to deal with frustration, disappointment and stress in their academic life flexibly. Students are able to internalize negative emotions into certain positive cues, thus avoiding the phenomenon of learning burnout at the source to a greater extent and are less likely to be disturbed by negative learning emotions such as anxiety, irritability, and powerlessness (70). For this reason, this group of students is more able to achieve high grades and correspondingly increase their sense of academic achievement and self-confidence. The lower the level of smartphone dependence of university students, the better they are able to engage in their studies and the higher their level of academic adaptability. At the same time, the better they adapt to their studies, the more they feel a sense of achievement and self-confidence, and the lower their level of learning burnout.

### 4.5. Moderating effect of self-efficacy

This study found that as self-efficacy increased, the negative prediction level of academic adaptability on learning burnout was higher, i.e., the inhibitory effect of academic adaptability on learning burnout was stronger for university students with high self-efficacy compared to the group of university students with low self-efficacy. Self-efficacy moderates the relationship between academic adaptability and learning burnout. This suggests that in order to effectively reduce learning burnout, increasing the self-efficacy of university students at the same level of academic adaptability is not a less effective measure. Based on our study, we have found that self-efficacy helps learners take part in activities outside their comfort zone and serves as a stable psychological resource capital that encourages learners to learn to persevere in difficult situations. As a result, students with high self-efficacy were able to cope more adequately with challenges and had more immediate and successful learning experiences, thus increasing their learning resilience. This is consistent with the previous view (43). Moreover, this group of students has higher expectations of their development goals, they are able to manage the different learning resources at their disposal and cope with a wide range of situations. For this reason, they have higher academic adaptability (71). In addition, the higher the self-efficacy, the stronger its buffering effect on stress regulation. The higher the self-efficacy of students, the more effective they are at managing their emotions, which means that they have the ability to export negative emotions into positive affective feedback and are less likely to relent to the learning task at hand due to external stimuli, thus effectively reducing the emergence of learning burnout (45). Conversely, students with low self-efficacy are prone to low academic performance in their academic life. When they perceive themselves as incompetent, frustration or stress about their academic performance can force them to engage in avoidance behaviors, such as abandoning classroom activities (72). Students with high self-efficacy tends to have an optimistic attitude and readjust themselves when they encounter difficulties and adversities, thus facilitating the achievement of their goals and reducing their learning burnout (36). University students with high self-efficacy hold a more optimistic mindset and are better able to plan and manage their study life. They adapt well to their university studies, which means they are able to complete their academic tasks better and achieve better grades, gaining a higher sense of accomplishment, which in turn reduces their learning burnout. It is important to note that some students, lacking insight in terms of their overall personal level, are overconfident due to ignorance, inducing a higher sense of self-efficacy than their actual ability to perform and accomplish. Students who are influenced by a “prepare for the worst” mindset and use avoiding trouble and protecting themselves from disappointing negative outcomes or feedback as a reason not to demonstrate self-efficacy (73). Therefore, in regarding increasing students’ self-efficacy, these circumstances should be taken into account.

## 5. Research value

### 5.1. Contributions

This study revealed the mediating process of smartphone dependence affecting learning burnout from the perspective of academic adaptability, and verified the moderating role of self-efficacy in it, which has some reference value for the theoretical construction of the mechanism of learning burnout occurrence. There have been many studies related to the negative effects caused by smartphone dependence (14, 74, 75), which have explored the relationship between smartphone dependence and sleep and academic performance (30), shyness (76), self-esteem (77), and cognitive absorption and social networking services (78). Some other studies focus on academic adaptability and learning burnout (27, 64), self-efficacy and academic adaptability (41, 79, 80) and self-efficacy and learning burnout (26, 69). The correlation between smartphone dependence and learning burnout among university students have also been highlighted (28, 29, 81). Some researches have analyzed the moderating variables between the two. For example, the mediating effect of psychological capital (82) and the moderation effects of resilience (83) in this process are studied. However, little has been done to explore the mediating and moderating roles of both academic adaptability and self-efficacy in the effects of smartphone dependence on learning burnout. The present study confirms that academic adaptability and self-efficacy play a protective role in the process of smartphone dependence’s influence on learning burnout. Therefore, this study can enrich the theoretical research related to the relationship between smartphone dependence and learning burnout. In addition, the results of this study can provide practical and effective operational suggestions for university educators to prevent and intervene university students’ learning burnout, so as to reduce the level of university students’ learning burnout.

### 5.2. Limitations

Although this study explored the mechanism of smartphone dependence on university students’ learning burnout in a systematic way, the study still has certain limitations. First, the sample was limited by the source of cross-sectional data, and the confirmatory nature of the causal inference of the variables was still inadequate. Secondly, there may be selection bias and potential threats in the case of convenience sampling. Thirdly, in the Chinese version of the Smartphone Dependence Questionnaire, we use “phone” instead of “smartphone” to express related items. Although Chinese university students’ first impression of phone was smartphone, and they were told before filling in the questionnaire that “phone” referred to “smartphone” they used nowadays, it would be better to change “phone” instead of “smartphone” in the questionnaire in order to avoid students’ misunderstanding of the word “phone.” Finally, although the study overall elaborated the impact of smartphone dependence on Chinese university students’ learning burnout, it did not compare the specific situation of university students from different academic levels, university types, and regions with different economic development levels, which are due to the limitations of the authors’ time and energy. Therefore, as part of future research, follow-up studies should be designed and implemented using multiple data collection methods. Future studies could use longitudinal data to verify the causal relationships of the variables of interest and to compare university students from different academic levels, university types, and regions with different economic development levels.

## Data availability statement

The original contributions presented in the study are included in the article/Supplementary material, further inquiries can be directed to the corresponding authors.

## Ethics statement

The studies involving human participants were reviewed and approved by the Committee of Xiamen Institute of Technology. The patients/participants provided their written informed consent to participate in this study.

## Author contributions

CC designed the study and wrote the manuscript. YS analyzed the data. FX, CC, and JN collected the data. JN and YZ modified the manuscript. FX supervised the development of research and provided the funding support. All authors contributed to the article and approved the submitted version.

## Funding

This study received funding from Fujian Province College Counselors Research Project (Project No. JSZF2020070).

## Conflict of interest

The authors declare that the research was conducted in the absence of any commercial or financial relationships that could be construed as a potential conflict of interest.

## Publisher’s note

All claims expressed in this article are solely those of the authors and do not necessarily represent those of their affiliated organizations, or those of the publisher, the editors and the reviewers. Any product that may be evaluated in this article, or claim that may be made by its manufacturer, is not guaranteed or endorsed by the publisher.
