# Supplementary material for: The effect of smartphone dependence on learning burnout among undergraduates: the mediating effect of academic adaptability and the moderating effect of self-efficacy
Source: Front Psychiatry. 2023 Sep 6;14:1155544. doi: 10.3389/fpsyt.2023.1155544 (PMC10509764; doi:10.3389/fpsyt.2023.1155544)
Supplement: Supplementary file 1 [file Data_Sheet_1.doc]

**APPENDIX (The scales used in the study are all validated authoritative scales.)**

1. **Toda, M., Monden, K., Kubo, K., & Morimoto, K. (2006). Mobile Phone Dependence and Health-Related Lifestyle of University Students. *Social Behavior and Personality: An International Journal*, 34(10), 1277–1284.**

Note: Based on the needs of this study and the reality, we have changed "mobile phone dependence" to "smartphone dependence" in the revised questionnaire. The revised questionnaire was conducted in Chinese, not English, so this translation is more in line with the students’ understanding of what they were being asked to consider in completing the questionnaire.”

**SMARTPHONE DEPENDENCE QUESTIONNAIRE**

1. I give my smartphone more priority than clothes and food.

2. I feel unsettled when I forget to take my smartphone with me.

3. I would rather lose my wallet or purse than my smartphone.

4. I recharge my smartphone battery every day.

5. I don’t really want to go to places where smartphone signals are weak.

6. When I am riding on a train or in similar situations, I tend to handle my smartphone.

7. Even while riding on trains, I make and receive calls.

8. I use my phone when I am in the company of one or two other people.

9. I make smartphone calls even late at night.

10. I talk on my smartphone for more than one hour a day.

11. I find it hard to keep company with people who don’t have smartphones.

12. Without thinking, I check my phone for email or voice mail even when it hasn’t rung.

13. I send mail even when I am at work or in class.

14. I send ten or more emails a day.

15. I am pleased when I receive email.

16. I send mail with little content that has no practical purpose.

17. I use a lot of pictographs in my email

18. I always reply to phone email.

19. I send lots of long email messages.

20. I express my true feelings better via email than by voice mail.

Respondents were asked to score each item as follows:

Always, 3 points;

Often, 2 points;

Sometimes, 1 points; or

Hardly ever, 0 points.

The total score indicates the level of smartphone dependence.

**Feng TY, Su Ti, Hu XW, Li H. The development of a test about learning adjustment of undergraduate. *Journal of psychology.* 2006 (5): 762-769.**

Learning Adaptation Scale for undergraduate Students" compiled by Feng Tingyong et al.

1. I think college students should not only learn knowledge, but also develop social skills.
2. I feel that I adapt to the study in university.
3. I always feel uncomfortable with the teaching style of university teachers.
4. I have my own study methods and plans, and I can put them into practice.
5. I don't adapt to the work and rest time of the university.
6. I feel that I have lost my learning goal.
7. After going to college, my way of thinking is more mature.
8. There is too much free time in college.
9. The campus is not in good order, and the lack of security affects my study.
10. I think I have more flexibility after I go to college.
11. My motivation to study is affected because I am not interested in my major courses.
12. The economic condition of my family has a great influence on my study.
13. My independence has increased significantly after I went to college.
14. I don't like to participate in various cultural and sports activities in school.
15. The bad opinion of the society about college students (such as "studying is useless") causes me to waste my study.
16. After I go to college, I become lazy.
17. I feel that after going to university, I have a broader understanding and a clearer future.
18. Study in college is only based on personal interest, no need for any method.
19. In the face of the fierce competition in college, I always try to improve myself.
20. I feel very uncomfortable with the disconnection between university study and secondary school.
21. The way students are managed in college is not as good as in high school.
22. I am annoyed when I mention studying.
23. After I go to college, I get into a lot of bad habits.
24. I would have stopped studying long ago if not for the credits and diploma.
25. I often miss my former classmates and things and can't help myself.
26. I don't adapt to this kind of university education which is more theoretical and less practical.
27. After I go to college, my practical ability is obviously enhanced.
28. Why take it too seriously? Open one eye, close one eye, you will adapt.
29. Life conditions in college have a big influence on learning.
30. The future employment situation seriously affects my study.
31. How well you handle interpersonal relationships in college has a great impact on your studies.
32. I am not used to the indifferent attitude of university teachers toward students.
33. My learning is by integration, not by rote.
34. I don't know how to organize my time, and I have no sense of urgency in studying.
35. The study atmosphere of the university is very adaptable to me.
36. After I went to college, my study goals became more clear.
37. The university campus lacks counseling for students' study, life and psychology.
38. I can arrange my leisure time very well.
39. After I go to college, I mainly study on my own.
40. The excessive social activities in college affect my study.
41. My study is very effective.
42. I find it difficult to study and have no confidence at all.
43. I feel my knowledge is insufficient, so I study harder.
44. I think college is a place where people's will is worn out.

Divided into 5 levels: Fully conforming, Comparatively conforming, Uncertain, Comparatively not conforming, Completely not conforming.

**Wang YZ (Ed.). *Handbook of Psychological Rating Scale (1999-2010)*. Zhengzhou University Press (2011): 45-48.**

**The Learning Burnout Undergraduates Scales (LBUS) developed by Lian Rong et al.**

**Learning Burnout Undergraduates Scales (LBUS)**

1. I have my own study methods and plans, and I can put them into practice＊

2. I feel that the knowledge I have learned is useless

3. Mastering professional knowledge is easy for me＊

4. I get up early in the morning and feel tired when I think about the day of studying

5. It is difficult for me to stay enthusiastic about studying for a long time

6. I am able to handle my emotional problems calmly when studying＊

7. I feel exhausted after studying all day

8. So far, my university studies have enabled me to show my abilities to the fullest＊

9. I am bored with my studies

10. I rarely study after class

11. I can handle my university classes＊

12. I often fall asleep while studying

13. I am interested in my major＊

14. I don't think I have enough patience when it comes to studying

15. It's easy for me to get a bachelor's degree＊

16. I study only when I take exams

17. I want to study but I feel that studying is boring

18. I have a lot of energy when I study＊

19. I rarely plan my study time

20. Exams always bore me

**Note: 1,3,6,8,11,13,15,18 is reverse scoring.**

Divided into 5 levels: Fully conforming, Comparatively conforming, Uncertain, Comparatively not conforming, Completely not conforming.

1. **Schwarzer, R., Bäßler, J., Kwiatek, P., Schröder, K., & Zhang, J. X. The Assessment of Optimistic Self-beliefs: Comparison of the German, Spanish, and Chinese Versions of the General Self-efficacy Scale. *Applied Psychology. 1997* 46(1): 69–88.**

The Self-Efficacy Scale was revised by Schwarzer et al. to measure general self-efficacy with 10 questions with good reliability and validity.

There are four options: Not at all correct, Somewhat correct, Mostly correct, Completely correct.

1. If I do my best, I can always solve the problem.

2. Even if others oppose me, I still have the means to get what I want.

3. It's easy for me to stick to my ideals and reach my goals.

4. I am confident that I can effectively deal with anything that comes up unexpectedly.

5. With my talents, I can definitely handle the unexpected.

6. I can solve most problems if I put in the necessary effort.

7. I can face difficulties calmly because I trust my ability to deal with problems.

8. When faced with a problem, I can usually find several ways to solve it.

9. When there is trouble, I can usually think of some ways to deal with it.

10. No matter what happens to me, I can handle it well.
